# Common Ayurvedic, Chinese traditional and Unani antidiabetic formulations- a review

**DOI:** 10.3389/fphar.2022.991083

**Published:** 2022-10-12

**Authors:** Prajakta H. Murudkar, Mukul S. Tambe, S.B. Chandrasekar, Bhavani Boddeda, Anil T. Pawar

**Affiliations:** ^1^ School of Pharmacy, Dr. Vishwanath Karad MIT World Peace University, Pune, India; ^2^ Drug Testing Laboratory, Drug Control Department, Bangalore, India; ^3^ Koringa College of Pharmacy, East Godavari, India

**Keywords:** Diabetic complications, diabetes mellitus, hyperglycemia, metabolic disorder, polyherbal formulations

## Abstract

Diabetes mellitus is the most widely recognized endocrine disorder which is influencing a bigger populace on the planet. There are various causes of diabetes, such as physical inactivity, obesity, family history, race, and age. Diabetes mellitus is associated with some life-threatening complications, such as neuropathy, nephropathy, various eye diseases or retinopathy, and cardiovascular disorders. Many synthetic antihyperglycemic agents are available in the market for the treatment of diabetes and its complications. But, due to some serious side effects of these synthetic agents, people are opting for herbal remedies and, therefore, they are now becoming popular. Herbal remedies have lesser side effects and higher affordability and therefore can be preferably used over synthetic agents for a long-term disorder like diabetes mellitus. In the present study, scientific research and review studies on the topic were collected from Science Direct, Scopus, PubMed, Google Scholar, and other relevant sources. The references of all the articles were screened manually for any additional information on popular polyherbal formulations in traditional Ayurvedic, Chinese, and Unani medicinal systems. It is found that these polyherbal formulations are studied for anti-diabetic potential. Furthermore, some are also investigated for mechanism of action of anti-diabetic effects. This review highlights various Ayurvedic, Chinese, and Unani polyherbal formulations commonly utilized in the management of diabetes mellitus along with their pre-clinical and clinical investigations, which will enhance the existing knowledge of the researchers.

## Graphical Abstract

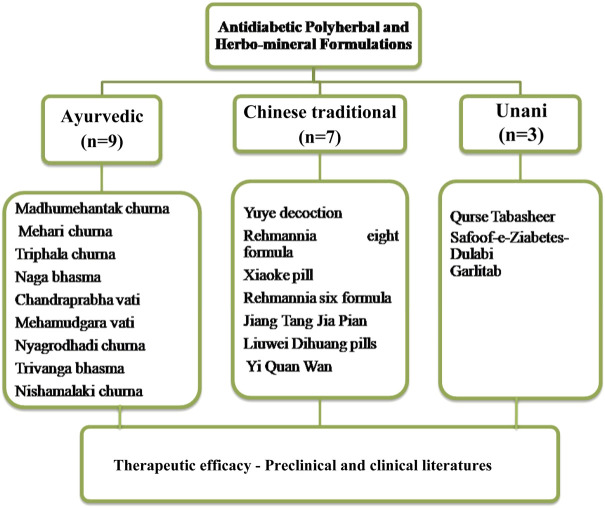

## Introduction

Diabetes mellitus is a disorder characterized by chronic hyperglycemia in which insulin secretion, insulin action, or both are defective (Kharroubi 2015). It causes life-threatening complications such as neuropathy, nephropathy, various eye diseases or retinopathy, cardiovascular disorders, etc., if left untreated. As per the World Health Organization, about 422 million of the global population, particularly in low- and middle-income countries, is suffering from diabetes mellitus. This high prevalence of diabetes is leading to an excessive burden on public medical service budgets. Diabetes is projected to be one of the principal life-threatening disorders of the globe within the next 25 years. There are various drugs, such as insulin preparations and oral hypoglycemic agents, available for treatment of diabetes mellitus. Despite these, numerous traditional and alternative therapies are popular among people for management of the diabetic condition (https://www.who.int/news-room/fact-sheets/detail/diabetes).

In traditional systems of medicine, various medicinal herbs and herbal-based formulations have been recognized and found to be effective for diabetes mellitus. These traditional herbal medicines have been commonly used due to their cost-effectiveness and lesser side effects (Nagappa et al., 2003). Countries with ancient civilizations, such as China, India, and Egypt, are still utilizing several herbal-based formulations for different health conditions, including diabetes mellitus (Sharma et al., 2008). These polyherbal formulations are safe and effective as compared to the single-herb formulation (Petchi et al., 2014). There is a curiosity among common people and researchers to obtain information about various herbal-based formulations used in traditional systems of medicines for the management of diabetes mellitus. With this background, the present review highlights pre-clinical and clinical aspects of various polyherbal formulations commonly used in Ayurveda, Chinese traditional and Unani systems of medicines for diabetes mellitus.

## Methods

Scientific research and reviews on the topic were collected from Science Direct, Scopus, PubMed, Google Scholar, and other sources. Articles from the journals indexed in Scopus, Web of Science, and UGC care list were selected for further study. Also, the references of all the articles were screened manually for any additional information on popular polyherbal and herbo-mineral formulations in traditional Ayurvedic, Chinese, and Unani medicine.

### Ayurvedic antidiabetic polyherbal formulations

In Ayurveda, single or multiple herbs (polyherbal) are utilized for treatment of health problems. The idea of polyherbalism was attributed to the Ayurvedic writing *Sarangdhar Samhita*. Individual plants and their phytoconstituents are insufficient to achieve desired beneficial effects. When multiple herbs are combined or polyherbal formulations are prepared or formulated in a particular ratio, they provide a better therapeutic effect and reduce the toxicity of herbs (Parasuraman et al., 2014).

In herbal preparations, a variety of therapeutic plants, known as *Rasayana*, have been used for over 1,000 years. Many Ayurveda practitioners formulate their own Ayurvedic formulations and dispense them to the patients for clinical benefits (Modak et al., 2007). Some of the commonly used Ayurvedic antidiabetic formulations are Madhumehantak churna, Mehari churna, Triphala churna, Naga bhasma, Chandraprabha vati, Mehamudgara vati, Nyagrodhadi churna, Trivanga bhasma, and Nishamalaki churna (Table 1).

**TABLE 1:** Summary of the various formulations of Ayurveda, Chinese, and Unani medicine systems used for the management of diabetes.

Formulation	Type of study	Dose and mode of administration	Outcome parameter	Improvement in diabetes	Reference
Madhumehantak churna	*In vivo* (clinical trial)	4 g thrice daily 30 min before food	Blood glucose level and HbA1c	Yes	Bhattacharya and Reddy (2016)
Mehari churna	*In vivo* (clinical trial)	1 to 2 teaspoonfuls with warm water	Blood glucose level and HbA1c	Yes	Kandalkar and Bhajipale (2016)
Triphala churna	*In vivo* (pre-clinical trial)	250, 500, and 1,000 mg/kg	Urine parameters, plasma proteins, albumin, creatinine, blood urea nitrogen levels, and oxidative stress in the kidney	Yes	Ganeshpurkar et al. (2015); Suryavanshi et al. (2020)
Naga bhasma	*In vivo* (pre-clinical study)	100 and 200 mg/kg	Weight deviation and glucose tolerance test	Yes	Deshmukh et al. (2013)
Chandraprabha vati	*In vivo* (pre-clinical study)	50, 100, and 200 mg/kg	Fasting glucose levels, glucose tolerance test, cholesterol, and triglycerides	Yes	Wanjari et al. (2016)
Mehamudgara vati	*In vivo* (clinical trial)	Three tablets thrice a day	Fasting blood sugar, postprandial blood sugar in blood and urine, serum cholesterol, triglyceride, and high-density lipoprotein	Yes	Tanna et al. (2011)
Nyagrodhadi churna	*In vivo* (clinical trial)	Two tablets thrice daily with lukewarm water	Fasting and post-prandial blood sugar, serum cholesterol, triglyceride, high-density lipoprotein, low-density lipoprotein, very low-density lipoprotein, fasting serum insulin, blood urea, and serum creatinine	Yes	Kumari et al. (2010)
Trivanga bhasma	*In vivo* (clinical trial)	200 mg/kg in water	Various blood parameters; fasting blood sugar	Yes	Das et al. (2018)
		200 mg/kg in honey			
Nishamalaki churna	*In vivo* (pre-clinical study)	900 and 1800 mg/kg	Blood glucose level	Yes	Dawane et al. (2016)
Yuye decoction	*In vivo* (pre-clinical study)	6.4, 12.7, and 25.4 g/kg	Fasting blood glucose, oral glucose tolerance, serum alanine aminotransferase, aspartate aminotransferase, creatinine, blood urea nitrogen, low-density lipoprotein, total cholesterol, triglyceride, high-density lipoprotein, number of islet cells, and the volume of islet cells	Yes	Liu et al. (2021)
Rehmannia Eight Formula	*In vivo* (pre-clinical study)	Twelve pills each time, three times daily	Blood glucose levels and lipid profile	Yes	Zhang et al. (2004)
Xiaoke Pill	*In vivo* (clinical trial)	Five pills per day	Blood glucose levels, HbA1C	Yes	Zhang et al. (2017)
Rehmannia Six Formula	*In vivo*	27 g/day	Frantic fever and spontaneous perspiration, total cholesterol, systolic blood pressure, waist circumference, phosphatidylcholines, and Yin deficiencies	Yes	Wietmarschen et al. (2009); Poon et al. (2011)
Jiang Tang Jia Pian	*In vivo* (clinical trial)	40 mg tablets thrice a day	Glucose tolerance	76.3%	Grant et al. (2009)
Liuwei Dihuang pills	*In vivo* (pre-clinical study)	2.4 g/kg	Diabetic nephropathy, fasting blood glucose, total cholesterol, triglycerides, low-density lipoprotein- cholesterol, HbA1C, urine volume levels urine sugar, and high-density lipoprotein- cholesterol level	Yes	Lin et al. (2016); Wang et al. (2018)
Yu Quan Wan	*In vivo*		Proinflammatory cytokines, blood glucose levels, and glucose tolerance	Yes	Grant et al. (2009)
Qurse Tabasheer					Shaikh et al. (2016)
Safoof-e-Ziabetes-Dulabi	*In vivo* (pre-clinical study)	200 and 400 mg/kg	Serum alkaline phosphatase, total bilirubin, serum glutamic pyruvic transaminase, and serum glutamic oxaloacetic transaminase	Yes	Syed Hussain et al. (2020)
Garlitab	*In vivo* (pre-clinical study)	1.25 mg/kg once daily	Blood glucose level	Yes	Hossain, (2015)

### Madhumehantak churna

The Madhumehantak churna (MMC) is one of Ayurvedic polyherbal formulations. This formulation is a combination of eleven herbs individually having reported antidiabetic activity, namely, amra-beej-majja (*Mangifera indica* Linn. seed) (Ganogpichayagrai et al., 2017), karavella phalli (*Momordica charantia* Linn. fruits) (Welihinda et al., 1986), jambubeej (*Syzygium cumini* Linn. fruits) (Kumar et al., 2008), nimbabeej (*Azadirachta indica* Linn. seeds) (Patil et al., 2013), palandubeej (*Allium sepa* Linn. seeds) (Mathew and Augusti 1975), babbula phalli (*Acacia nilotica* Linn. fruits) (Ahmad et al., 2008), balabeej (*Sida cordifolia* Linn. seeds) (Kaur et al., 2011), Methikabeej (*Trigonella foenum-graecum* Linn. fruits) (Ghosh et al., 2009), meshashrungi (*Gymnema sylvestre* R. Br.) (Baskaran et al., 1990), haridra (*Curcuma longa* Linn.) (Lekshmi et al., 2014), and haritaki-phala-majja (*Terminalia chebula* R. fruits) (Borgohain et al., 2012; Bhattacharya and Reddy 2016).

An experimental preclinical study was performed on laboratory animals. Diabetic rats were treated with MMC for 28 days to prove that MMC is safe and effective. The effect of MMC on blood glucose showed a lowering of blood glucose effectively at two doses, 216 and 648 mg/kg, when compared with diabetic control group. The dose of 216 mg daily to rats is equivalent to 1 karsa ∼12 g dose, recommended by Ayurveda as an optimal human dose (Bhattacharya and Reddy 2016).

A clinical study was also performed with a total of 101 out-patients placed into three groups. They were treated with MMC for three continuous months, preceded and followed by an assessment of long-term glucose control using glycated hemoglobin (HbA1c) testing. The MMC was found effective in lowering blood glucose and HbA1c in diabetic patients (Bhattacharya and Reddy 2016).

### Mehari churna

Mehari churna is a commonly used antidiabetic polyherbal formulation in Ayurveda. This formulation is a powder of various herbs helpful in treating pre-diabetes conditions and diabetes mellitus. It consists of various Ayurvedic ingredients possessing antidiabetic potentials like turmeric (*Curcuma longa* Linn.) (Kuroda et al., 2005), amla (*Emblica officinalis* Gaertn.) (Akhtar et al., 2011), jamun (*Syzygium cumini* Linn.) (Kandalkar and Bhajipale 2016), guduchi (*Tinospora cordifolia* Thunb. Miers) (Patel and Mishra 2011), manjishtha (*Rubia cordifolia* Linn.) (Rani et al., 2013), methi (*Trigonella foenum-gracecum* Linn.) (Al-Juraisy 2014), and hirda (*Terminalia chebula* R.) (Borgohain et al., 2012).

Mehari churna helps to reduce symptoms of diabetes. It normalizes the blood glucose level by enhancing insulin release from the beta cells of the pancreas and thereby prevents diabetes and diabetic complications. Mehari churna stimulates pancreatic action and improves the release of insulin. This churna also improves the overall lipid profile by lowering low-density lipoprotein or low-density cholesterol. It also reduces excessive urination and glycosuria (Kandalkar and Bhajipale 2016).

### Triphala churna

Triphala churna is a well-recognized and highly efficacious Ayurvedic polyherbal formulation. It consists of fruits of the antidiabetic plants, namely, amla (*Emblica officinalis* Gaertn.) (Akhtar et al., 2011)*,* beheda (*Terminalia bellerica* gaertn. Roxb) (Gupta et al., 2020), and hirda (*Terminalia chebula* R.) (Borgohain et al., 2012). Triphala churna is an antioxidant-rich polyherbal formulation having diverse beneficial properties. As per the Ayurvedic Formulary of India (AFI), Triphala churna is prepared by mixing amla, behda, and hirda in a ratio of 1:1:1. Triphala churna has immune-modulatory properties and, therefore, it helps in improving the body’s defense system (Prativadibhayankaram et al., 2008).

Triphala churna has antidiabetic activity and also treats some of its complications. It is evaluated for diabetic nephropathy in streptozotocin-induced diabetic rats. The diabetic rats were treated with Triphala churna at doses of 250, 500, and 1,000 mg/kg for 4 weeks. Results showed that treatment with Triphala churna significantly improves urine parameters. It also caused improvement in various biochemical parameters, such as plasma proteins, albumin, creatinine, and blood urea nitrogen levels. After treatment with Triphala churna, oxidative stress was reduced in the kidney. Histopathology showed that Triphala churna decreases renal or kidney damage in experimental rats and helps in treating diabetic nephropathy (Ganeshpurkar et al., 2015; Suryavanshi et al., 2020).

### Naga bhasma

Naga bhasma is an ancient Ayurvedic herbo-mineral formulation. It contains Naga which is also known as calcinated lead. The complex found in Naga bhasma is known as lead sulfide. This lead sulfide is mixed with plant materials and may contain a variety of mineral deposits of plants. Antidiabetic properties of Naga bhasma were found around the 12th century CE. Naga bhasma mainly contains antidiabetic constituents, namely, haridra (*Curcuma longa* L.) (Kuroda et al., 2005), amalaki (*Emblica officinalis* Gaertn.) (Akhtar et al., 2011), guduchi (*Tinospora cordifolia* (Thunb.) Miers) (Patel and Mishra 2011), and madhu (honey) (Hemmati et al., 2015). Naga bhasma, therefore, shows antidiabetic activity and is also capable of treating diabetic complications (Tate et al., 2009).

A preclinical study was carried out for assessment of the antidiabetic effect of Naga bhasma in alloxan-induced diabetic rats. Naga bhasma was given at a dose of 100 and 200 mg/kg for 14 days in the form of suspension with milk by oral gavage to the normal and alloxan-induced diabetic rats. Glucose tolerance test was conducted in experimental rats. In this study, glibenclamide was used as a standard drug. It was observed that treatment with Naga bhasma regularized the impaired glucose tolerance on long-term treatment. It was, therefore, concluded that Naga bhasma has an antidiabetic effect and also treats diabetic complications (Tate et al., 2009; Deshmukh et al., 2013).

### Chandraprabha vati

Chandraprabha vati is an ancient Ayurvedic polyherbal formulation. This formulation is notably utilized for improvement of *Prameha* i.e., diabetes (Baskaran et al., 1990). This formulation is known to have potent anti-inflammatory properties and is also used to treat diseases of the urinary tract, thyroid gland, kidney, pancreas, bones, and joints. It contains thirty-seven herbo-mineral ingredients. Many of these ingredients such as *Acorus calamus* Linn. (Prisilla et al., 2012), *Cyperus rotundus* Linn. (Singh et al., 2015), *Phyllanthus niruri* Linn. (Okoli et al., 2010), *Tinospora cordifolia* Thunb. Miers (Patel and Mishra 2011), *Curcuma longa* Linn. (Lekshmi et al., 2014), *Berberis aristata* DC. (Upwar et al., 2011), *Piper longum* Linn. (Nabi et al., 2013), *Coriandrum sativum* Linn. (Naquvi et al., 2004), *Terminalia chebula* Retz. (Borgohain et al., 2012), *Terminalia belerica* Gaertn. Roxb. (Gupta et al., 2020), *Embelica officinalis* Gaertn. (Akhtar et al., 2011), *Embelia ribes* Burm. f. (Bhandari et al., 2013), *Zingiber officinale* Roscoe. (Al-Amin et al., 2006), *Piper nigrum* Linn. (Kaleem et al., 2005), *Hordeum vulgare* Linn. (Deng et al., 2020), *Ipomoea turpethum* Linn. (Pulipaka et al., 2012), and *Cinnamomum zeylanicum* Linn. (Verspohl et al., 2005) showed remarkable antidiabetic effects in several studies.

The antihyperglycemic effect of Chandraprabha vati was studied in Wistar rats. This formulation was administered at the doses of 50, 100 and 200 mg/kg orally for 7 days to alloxan-induced diabetic rats. An oral glucose tolerance test was also performed. Fasting glucose levels were determined at different time intervals. Cholesterol and triglyceride levels were also measured. Metformin (500 mg/kg orally) was used as a standard drug (Tate et al., 2009). It was concluded that Chandraprabha vati has an antihyperglycemic and antihyperlipidemic effects. The findings support the use of Chandraprabha vati in clinical practice for diabetes mallitus (Wanjari et al., 2016).

### Mehamudgara vati

The Mehamudgara vati is an Ayurvedic herbo-mineral formulation containing Lauha bhasma, guggulu (*Commiphora wightii* (Arnott) Wightii), haritaki (*Terminalia chebula* R.), bibhitaki (*Terminalia bellerica* Gaertn. Roxb), amalaki (*Embelica officinalis* Gaertn.), shunthi (*Zingiber officinalis* Roscoe.), marich (*Piper nigrum* Linn.), pippali (*Piper longum* Linn.), trivrita (*Operculina terpethum* L.), pippalimula (*Piper longum* Linn.), *Bidalavana*, bilva (*Aegle marmelos* Linn.), gokshura (*Tribulus terrestris* Linn.), dadima (*Punica granatum* Linn.), devadaru (*Cedrus deodara* Roxb. G.Don), rasanjana (*Berberis aristata* DC.), Kiratatikta (*Swertia chirayita* Linn.), and Triphala kwatha. Mehamudgara vati is well known for its hypolipidemic, hypocholesterolemic, antihyperglycemic, and antioxidant activities, which make this medicine beneficial for diabetic patients.

The clinical efficacy of Mehamudgara vati (MMV) was studied in type 2 diabetic patients (Tanna et al., 2011). In this study, one group of patients with mild-to-moderate type 2 diabetes was given three tablets of MMV, thrice a day for 3 months. In another group, the type 2 diabetic patients who took modern antidiabetic treatment but with uncontrolled diabetes were additionally given MMV in the same manner. The MMV was found to be highly effective in decreasing both fasting as well as postprandial blood sugar levels. Furthermore, it is established that MMV has synergistic action when combined with modern antidiabetic drugs (Tanna et al., 2011).

### Nyagrodhadi churna

Chakradatta, an Ayurvedic text, mentions Nyagrodhadi churna, which is converted into ghanvati for improving patient compliance. This formulation contains antidiabetic plants namely vata (*Ficus benghalensis* Linn.)*,* udumber *(Ficus racemosa* Linn.)*,* aswatha *(Ficus religiosa* Linn.*),* bijaka *(Pterocarpus marsupium* f.), Amra *(Magnifera indica* Linn.*),* jambu *(Syzygium samarangense* Blume.*),* arjuna *(Terminalia arjuna* Roxb.*),* dhava *(Anogeissus latifolia* Roxb.), paribhadra *(Erythrina orientalis* Murr.*), meshashringi (Gymnema sylvestre* R. Br.), chitrak (*Plumbago zeylanica* Linn.*),* karanja *(Millettia pinnata* Linn.*),* triphala, kutaja *(Holarrhena pubescens* Wall.), and bhallataka *(Semecarpus anacardium* Linn.). Amalaki (*Emblica officinalis* Gaertn.), a constituent of Nyagrodhadi churna, is an immunomodulator along with an antihyperglycemic effect and thus may be used in autoimmune diabetes mellitus (Kumari et al., 2010). Similar effects are found in bhallataka *(Semecarpus anacardium* Linn.*),* haritaki (*Terminalia chebula* Retz.)*,* mulethi (*Glycyrrhiza glabra* Linn.), madhuka *(Madhuca longifolia* J. Konig.), dhava *(Anogeissus latifolia* Roxb.*)*, etc. The herbs like bijaka *(Pterocarpus marsupium* f.)*,* arjuna *(Terminalia arjuna* Roxb.*),* chitraka *(Plumbago zeylanica* Linn.*),* patola *(Cucumis acutangulus* Linn.*),* meshashringi *(Gymnemasylvestre* R. Br.*),* amalaki (*Emblica officinalis* Gaertn.)*,* haritaki (*Terminalia chebula* Retz.), etc. have a hypolipidemic action (Simha and Laxminarayana, 2007; Kumari et al., 2010).

### Trivanga bhasma

Trivanga bhasma (TB) is a metallic preparation containing bhasmas of *Naga*, *Vanga*, and *Yashada*, i.e., lead, tin, and zinc, respectively (Jamadagni et al., 2017). Sprague Dawley rats were injected with streptozotocin (STZ) to induce diabetes followed by dosing of drugs (TB with herbal ingredients (bhawna) and without herbal ingredients) in a human equivalent therapeutic dose i.e., 200 mg/kg for 30 days. Various blood parameters and histopathology of organs were analyzed to evaluate the antidiabetic effect and reveal the side effects of the drugs. TB with herbal ingredients showed better antidiabetic effects in STZ-induced diabetic rats. In the prophylactic study, Trivanga bhasma-treated animals had lower blood sugar levels than non-treated rats even after injecting them with STZ. After treatment with TB for 30 days, the fasting blood sugar level of diabetic animals was significantly lower than that of diabetic control animals in a curative study (Das et al., 2018).

### Nishamalaki churna

This herbal preparation contains two ingredients amla (*Emblica officinalis* Gaertn.) and turmeric (*Curcuma longa L.*)*.* Amla and turmeric are mixed in equal parts to prepare Nishamalaki churna. In Ayurveda, Nishamalaki churna was recommended to treat the initial phase of diabetes mellitus. This formulation also possesses antioxidant and neuroprotective properties (Das et al., 2018).

A preclinical study was carried out to determine the antidiabetic effect of Nishamalaki churna. In this study, diabetes was induced by intraperitoneal administration of streptozotocin at a dose of 35 mg/kg to the rats. A high-fat, high-fructose diet was also given along with streptozotocin injection. The diabetic animals were treated with Nishamalaki churna at a dose of 900 and 1800 mg/kg for 12 weeks and blood glucose levels were determined after 15 days. Rats were also examined for their lipid profile. For determination of thermal hyperalgesia and cold allodynia, methods like Eddy’s hot plate and tail immersion method were used. Results of the study showed that diabetic rats treated with Nishamalaki churna have an improvement in thermal hyperalgesia and cold allodynia. Nishamalaki churna was found to lower blood sugar levels and thus possesses anti-diabetic activity (Dawane et al., 2016).

### Chinese antidiabetic polyherbal formulations

Traditional Chinese Medicine (TCM) is a holistic system of medicines that focuses on increasing the body’s resilience to illness. TCM uses herbal therapeutic strategies to improve health (Lu et al., 2004). Commonly used Chinese antidiabetic formulations are Yuye decoction, Rehmannia eight formula, Xiaoke pill, Rehmannia six formula, Jiang Tang Jia Pian, Liuwei Dihuang pills, and Yi Quan Wan (Table 1).

### Yuye decoction

Yuye decoction (YYD) is a traditional Chinese polyherbal formulation. This formulation was first mentioned in the customary Chinese medication volume called “yixuezhongzhongcanxilu”. This formulation is utilized in traditional Chinese medication to treat diabetes mellitus, essentially type 2 diabetes mellitus (Liu et al., 2021). There are seven medicinal herbs that compose Yuye decoction, namely, *Dioscorea* L, *Astragalus membranaceus* Fisch, *Trichosanthes kirilowii* Maxim*., Anemarrhena asphodgfoides* Bunge, *Schisandra chinensis* Turcz, *Gallus gallusdomesticus Brisson*, and *Pueraria lobata* Maesen.

For preclinical evaluation, rats with type 2 diabetes mellitus were used in the study (Liu et al., 2021). Diabetic condition was induced in rats by administration of high-fat, high-glucose diet for 6 weeks. Later, the diabetic rats were randomly divided into the diabetic group, the metformin (90 mg/kg) control group, and the modified *Yuye decoction* (6.4 g/kg, 12.7 g/kg, 25.4 g/kg) groups. The drug was administered for 4 weeks, and fasting blood glucose was monitored. After 4 weeks of treatment, oral glucose tolerance test was carried out. Results from fasting blood glucose and oral glucose tolerance test showed that modified *Yuye decoction* significantly improves the blood glucose levels and insulin resistance. In addition, modified *Yuye decoction* significantly reduced the serum levels of alanine aminotransferase, aspartate aminotransferase, creatinine, blood urea nitrogen, low-density lipoprotein, total cholesterol and triglycerides, whereas it increased the level of high-density lipoprotein. At the same time, hematoxylin–eosin staining of pancreas, liver and kidney tissues showed that modified *Yuye decoction* increases the number of islet cells, reduces the volume of islet cells, improves liver edema and inflammation, and improves renal glomerular hypertrophy (Liu et al., 2021).

### Rehmannia eight formula

Rehmannia Eight Formula is well known, Chinese traditional formulation. It consists of Chinese foxglove root (*Rehmannia glutinosa* Gaertn.) (Zhang et al., 2004), Japanese cornel fruit (*Cornus officinalis* Torr.) (Kim 2005), Chinese yam (*Dioscorea polystachya* Turcz.) (Maithili et al., 2011), poria mushroom (*Poriacocos sclerotium* Peck.) (Lee et al., 2014), horny goat weed (*Epimedium grandiflorum* C. Morren.), water plantain (*Alisma plantago* Linn.), astragalus (*Astragalus membranaceus* Fisch.) or moutan root bark (*Paeonia suffruticosa* Andrews.) (He et al., 2011) and Chinese cinnamon bark (*Cinnamomum cassia* L.).

*Rehmannia Eight Formula* has an antidiabetic effect due to various herbal constituents. It is also known for blood glucose and lipid-lowering properties. *Cornus officinalis* Torr. and *Alisma plantago* Linn. are ingredients in the formula, which help to maintain blood glucose levels and improve the lipid profile. Cinnamon in *Rehmannia Eight Formula* prevents diabetes by lowering fasting blood glucose levels (Zhang et al., 2004).

### Xiaoke pill

Xiaoke Pill is a polyherbal formulation utilized for management of type 2 diabetes mellitus (Zhang et al., 2017). Xiaoke Pill consists of *Radix Puerariae, Radix Rehmanniae, Radix Astragali, Fructus Schisandrae Sphenantherae, Rhizoma Dioscoreae, Radix Trichosanthis,* and *Stylus Zeae Maydis.* The clinical outcome of Xiaoke Pill showed that it has greater glucose-lowering property (Zhang et al., 2017).

### Rehmannia six formula

This Chinese formula is used for the treatment of diabetes mellitus. There are a total of six Chinese herbs in this formulation, i.e*.*, *Rehmannia glutinosa* Gaertn. (Zhang et al., 2004), *Fructus corni (Cornus officinalis* Sieb.*)* (Kim 2005)*, Dioscorea opposita* Linn. (Maithili et al., 2011)*,* Poriacocos (*Wolfiporia extensa* Peck.) (Lee et al., 2014)*, Alisma orientalis* Sam, and *Paeoniasuf fruticosa* Andrews.

For the assessment of Rehmannia Six Formula, a clinical study was conducted in which various clinical parameters were measured. Symptoms like hectic fever and spontaneous perspiration were observed to be alleviated during this treatment time (Wietmarschen et al., 2009). There are evidences that plasma glucose levels of rats was reduced by treatment of this formulation but in humans, it did not show any effects on plasma glucose levels (Poon et al., 2011). Total cholesterol, systolic blood pressure, and waist circumference were also lowered (Wietmarschen et al., 2009).

### Jiang Tang Jia Pian

Jiang Tang Jia Pian is used in Chinese traditional medicine to treat diabetes mellitus. This formulation contains various Chinese herbs, namely, astragalus (*Astragalus membranaceus* Fisch.) (Yin et al., 2009), polygonatum (*Polygonatum kingianum* Mill.) (Lu et al., 2016), trichosanthes root (Zhang et al., 2004), pseudostellaria (*Pseudostellaria heterophylla R.*) (Hu et al., 2013), and rehmannia (*Rehmannia glutinosa* Gaertn.) (Yin et al., 2009). This formula is mainly suggested for patients who are capable of producing low insulin (Zhang et al., 2004). Clinical studies were performed to check the efficacy of Jiang Tang Jia Pian. The results showed that Jiang Tang Jia Pian can be considered for people with impaired glucose tolerance and it also reduces the incidence of diabetes (Grant et al., 2009).

### Liuwei Dihuang pills

Liuwei Dihuang pill formula in is a Chinese traditional medicine for treatment of diabetic nephropathy and also used as a dietary supplement (Lin et al., 2016). It contains Rehmannia glutinosa (Gaertn.) Steud, *Cornus officinalis* Torr, *Paeonia suffruticosa* Andrews, *Dioscorea opposite* Thunb, *Poriacocos* Fr., and *Alisma orientalis* Sam.

Clinical study was performed in order to check the effect of Liuwei Dihuang pills. The result showed that Liuwei Dihuang pills when given daily to the patients with diabetic nephropathy causes improvement in kidney function (Lin et al., 2016; Wang et al., 2018).

### Yu Quan Wan

This traditional Chinese formula is used to treat diabetes patients at a dose of 50 gm daily. This formulation is also known as Jade Spring Pills. It consist of various herbal Chinese ingredients, such as licorice (*Glycerrhiza glabra* Linn.) (Takii et al., 2001), pueraria (Wu et al., 2013), schizandra (*Schizandra repanda* Linn.) (Wu et al., 2013), and trichosanthes roots (*Trichosanthes cucumerinia* Linn*.*) (Arawwawala et al., 2009). It is helpful in the proper functioning of islets of Langerhans and are therefore used to treat early-phase diabetes. Preclinical studies showed that Yu Quan Wan improves liver glycogen levels. In clinical studies, Yu Quan Wan improved proinflammatory cytokines in patients with type 2 diabetes mellitus. It has significant glucose-lowering property and is thus recommended for treatment of diabetes mellitus (Grant et al., 2009).

### Unani antidiabetic polyherbal formulations

The Unani System of medicine is Greco–Arabic medicine, which is based on the knowledge of Greek and Roman physicians. It is not only the original science of medicine but also a rich storehouse of principles and philosophies of medicine that can be of immense value to medicine in general and science in particular (Ansari et al., 2017). Some of the Unani antidiabetic formulations are Qurse Tabasheer, , Safoof-e-Ziabetes-Dulabi, and Garlitab (Table 1).

### Qurse Tabasheer

Qurse Tabasheer contains six ingredients, namely, Tabasheer (Siliceous concretions) (*Bambosa arundinaceae* Retz.) (Jayarambabu et al., 2021), GuleSurkh (*Rosa damascena Mill*. flower) (Gholamhoseinian and Fallah 2009), Gulnar (*Punica granatum* Linn. flower) (Li et al., 2009), Tukhmekahu (*Lactucasatisva* Linn. seeds) (Bagri et al., 2009), Tukhmekhurfa (*Portulaca oleraceae* Linn. seeds) (Li et al., 2009), and Gile Armani (*Armenian bole*) (Ahmed et al., 2013). This particular formulation is mentioned in Unani texts, *Bayaaze Kabeer* and *Kitabul Murakkabat Al Maroof Makhzanul Murakkabat*. It is used in the treatment of *Dhayabitus* (diabetes), Hummae Hadda (acute fever), and Is’hal (diarrhea). Moreover, this formulation has been reported to possess hypoglycemic activity (Shaikh et al., 2016).

### Safoof-e-Ziabetes Dulabi

Safoof-e-Ziabetes-Dulabi is an Unani polyherbal preparation widely given for Ziabetussadiq and Zof-e-kulya. Ziabetussadiq is known as diabetes mellitus, and Zof-e-kulya is known as kidney disease. This formulation consists of six herbal ingredients, namely, Gular (*Ficus racemosa Linn*.) (Veerapur et al., 2012), Gulnarfarsi (*Punica granatum* Linn) (Bagri et al., 2009), Dana anarshireen (*Punica granatum* Linn.), Maghz-eTukhm-e-Anba (Aderibigbe et al., 2001), Kishneez Khushk (Naquvi et al., 2006), and Gil-e-Armani alongwith Qand Safaid (sugar). This preparation has antidiabetic, anticancer, and immunomodulatory activities.

An acute oral toxicity study of Safoof-e-Ziabetes-Dulabi (SZD) was conducted on Swiss albino mice and, the result of the toxicity study showed no toxicity at a dose of 2,000 mg/kg (Syed Hussain et al., 2020). The antidiabetic activity of SZD was evaluated in streptozotocin-induced diabetic rats. Various biochemical parameters were evaluated in the study. It was observed that when diabetic rats were given SZD at doses of 200 mg/kg and 400 mg/kg for 8 weeks, their body weight increased significantly as compared to that of the diabetic control group. It was also observed that the blood glucose levels and glycated hemoglobin were reduced in SZD-treated groups when compared with diabetic control. A liver function test was also performed to evaluate the safety profile of SZD. Treatment with SZD at 200 and 400 mg/kg for 8 weeks caused significant decrease in serum levels of alkaline phosphatase, total bilirubin, serum glutamic pyruvic transaminase (SGPT), and serum glutamic oxaloacetic transaminase (SGOT) when compared with the diabetic control group. Thus, the results suggested that SZD possesses antidiabetic activity (Syed Hussain et al., 2020).

### Garlitab

Garlic is the most recommended Unani medicine for health benefits. This formulation consists of garlic (*Allium sativum* L.) (Eidi et al., 2006), onion (*Allium sepa* L.) (Mathew and Augusti 1975), and clove (*Syzygium aromaticum* L.) (Kuroda et al., 2015). It also contains other constituents such as allicin, volatile oil, minerals, and vitamins, which are essential for good health (Hossain 2015).

In a preclinical study, the efficacy of Garlitab was evaluated. A formulation of garlic, onion, and clove was evaluated in streptozotocin-induced diabetic rats. The blood glucose level and body weight were measured. In this study, diabetic rats were treated with Garlitab at the dose of 1.25 mg/kg once daily for 28 days. The outcome of the study showed that Garlitab reduces blood glucose levels and therefore supports the traditional use of Garlitab as a hypoglycemic formulation (Hossain 2015).

## Conclusion

Diabetes has become a common health problem around the world in recent years, affecting people of all ages, genders, and races. Furthermore, its incidences have been increasing at an alarming rate day by day. The available synthetic antidiabetic medications cause substantial side effects. Many of the drugs cause damage or failure of vital organs like kidneys and the liver over a period of time. The use of multiple drugs for long duration results in the patient's non-compliance. As a result, a diverse range of traditionally claimed herbs have been used either exclusively or in the form of polyherbal formulations to manage diabetes and associated complications. These polyherbal formulations have potential antidiabetic effects, and scientists have performed several preclinical and clinical studies to establish their safety and efficacy profile. One additional advantage of these formulations is that many of them have efficacy on diabetes and its complications as well. This makes them patient-friendly. The side effects from these formulations are much lesser than that of synthetic drugs, although an overdose can have certain side effects. The ever-increasing safe use of such formulations with proper guidance from physicians can lead to a decreased burden of diabetes, worldwide. However, there is a need to examine active components and their molecular interactions to comply regulatory standards of modern medicines.
